# Dynamic contrast-enhanced magnetic resonance imaging-based radiomics for the prediction of progression-free survival in advanced nasopharyngeal carcinoma

**DOI:** 10.3389/fonc.2022.955866

**Published:** 2022-10-13

**Authors:** Wen-zhu Li, Gang Wu, Tian-sheng Li, Gan-mian Dai, Yu-ting Liao, Qian-yu Yang, Feng Chen, Wei-yuan Huang

**Affiliations:** ^1^ Department of Radiology, Hainan General Hospital, Hainan Affiliated Hospital of Hainan Medical University, Haikou, China; ^2^ Department of Radiotherapy, Hainan General Hospital, Hainan Affiliated Hospital of Hainan Medical University, Haikou, China; ^3^ Department of Pharmaceutical Diagnostics, GE Healthcare, Guangzhou, China

**Keywords:** dynamic contrast-enhanced magnetic resonance imaging, nasopharyngeal carcinoma, nimotuzumab, progression-free survival, radiomics

## Abstract

To establish a multidimensional nomogram model for predicting progression-free survival (PFS) and risk stratification in patients with advanced nasopharyngeal carcinoma (NPC). This retrospective cross-sectional study included 156 patients with advanced NPC who underwent dynamic contrast-enhanced magnetic resonance imaging (DCE-MRI). Radiomic features were extracted from the efflux rate constant (*K^trans^*) and extracellular extravascular volume (*V_e_*) mapping derived from DCE-MRI. Least absolute shrinkage and selection operator (LASSO) Cox regression analysis was applied for feature selection. The Radscore was constructed using the selected features with their respective weights in the LASSO Cox regression analysis. A nomogram model combining the Radscore and clinical factors was built using multivariate Cox regression analysis. The C-index was used to assess the discrimination power of the Radscore and nomogram. The Kaplan–Meier method was used for survival analysis. Of the 360 radiomic features, 28 were selected (7, 6, and 15 features extracted from *K^trans^*, *Ve*, and *K^trans^* +*V_e_* images, respectively). The combined Radscore*_k_^trans^_+Ve_* (C-index, 0.703, 95% confidence interval [CI]: 0.571–0.836) showed higher efficacy in predicting the prognosis of advanced NPC than Radscore*_k_^trans^* (C-index, 0.693; 95% CI, 0.560–0.826) and Radscore*_Ve_* (C-index, 0.614; 95% CI, 0.481–0.746) did. Multivariable Cox regression analysis revealed clinical stage, T stage, and treatment with nimotuzumab as risk factors for PFS. The nomogram established by Radscore*_k_^trans^_+Ve_* and risk factors (C-index, 0.732; 95% CI: 0.599–0.864) was better than Radscore*_k_^trans^_+Ve_* in predicting PFS in patients with advanced NPC. A lower Radscore*_k_^trans^_+Ve_* (HR 3.5584, 95% CI 2.1341–5.933), lower clinical stage (hazard ratio [HR] 1.5982, 95% CI 0.5262–4.854), lower T stage (HR 1.4365, 95% CI 0.6745–3.060), and nimotuzumab (NTZ) treatment (HR 0.7879, 95% CI 0.4899–1.267) were associated with longer PFS. Kaplan–Meier analysis showed a lower PFS in the high-risk group than in the low-risk group (*p*<0.0001). The nomogram based on combined pretreatment DCE-MRI radiomics features, NTZ, and clinicopathological risk factors may be considered as a noninvasive imaging marker for predicting individual PFS in patients with advanced NPC.

## Introduction

Nasopharyngeal carcinoma (NPC) is an epithelial carcinoma with a distinct geographical distribution, mainly occurring in Southern China and Southeast Asia (1). More than 70% of patients are diagnosed with advanced-stage (III–IVB) NPC at presentation, owing to its nonspecific clinical symptoms and concealed anatomical location (2). The standard treatment for patients with advanced NPC is cisplatin-based concurrent chemoradiotherapy (CCRT) with or without induction chemotherapy (IC) according to National Comprehensive Cancer Network (NCCN) guidelines (3). However, locoregional recurrence and distant metastasis are the main causes of treatment failure in advanced NPC, with 5-year overall survival (OS) rates of 67–77%, and 2-year progression-free survival (PFS) rates of 72.9% after standard treatment (4–6). Thus, accurately identifying patients at high risks for locoregional recurrence and distant metastasis before treatment can help to determine the need for more aggressive treatments. Modifying chemoradiation protocols or using anti-epidermal growth factor receptor (EGFR) monoclonal antibodies and immunotherapy can reduce locoregional recurrence and distant metastasis in these patients (7, 8). Therefore, predicting the high risk of poor prognosis among patients and optimizing personalized treatment strategies are critical.

The tumor-node-metastasis (TNM) staging system is widely used to predict prognosis and facilitate treatment stratification in patients with NPC. However, this system may not be sufficiently precise, as patients with the same TNM staging show different therapeutic responses and clinical outcomes (9). While some clinical and histopathological biomarkers are associated with survival in NPC patients (10, 11), the clinical utility of these biomarkers is limited and unclear. Thus, an important challenge in clinical practice is defining effective and non-invasive biomarkers for prognosis to help in the selection of patients who can most benefit from specific treatments and predict the long-term therapeutic consequences.

Radiomics is a rapidly emerging field in medicine that transforms medical images into mineable high-dimensional quantitative features *via* a large number of automatically extracted data-characterization algorithms associated with tumor diagnosis, prognosis, and prediction of response to treatment (12, 13). In NPC, radiomic analysis from multiparametric MR images has been successfully performed to predict individual PFS in advanced NPC (4, 14). Moreover, the radiomics features of MR images are useful for predicting treatment response to chemoradiotherapy (15) and IC (16, 17) in patients with NPC. However, most previous studies focused on conventional MR sequences. Dynamic contrast-enhanced (DCE)-magnetic resonance imaging (MRI) is an MR perfusion technique that consists of a series of rapid contrast-enhanced T1-weighted acquisitions. With proper quantitative analysis, the data provides functional information on blood flow, vascular permeability, and angiogenesis, in addition to the morphological tumor characteristics used in clinical practice (18). Quantitative DCE-MRI parameters can be easily derived for each pixel within the tumor using commercially available software and can be visualized in a separate image for each parameter (19). This makes DCE-MRI a powerful prognostic tool. Our team also confirmed that DCE parameters can distinguish hypoxia-inducible factor-1α (HIF-1α), EGFR, and Ki-67 expression, which mediates prognosis in NPC tumors (20). Recent studies showed that DCE-MRI-based radiomics is more efficient than conventional MR sequences in predicting the prognosis and treatment response in malignant gliomas (21), breast cancer (22) and rectal cancer (23). To our knowledge, no published study has used DCE-MRI-based radiomics to predict individual PFS in patients with advanced NPC.

This study established a nomogram model combining radiomic features based on DCE-MRI, NTZ treatment, and clinical risk variables to predict individual PFS in patients with advanced NPC (stage III–IVB). Patients were divided into high- and low-risk groups based on the nomogram model to evaluate the model efficiency. This may provide a novel tool for risk stratification and individualized therapy protocols for NPC.

## Materials and methods

### Patients

The Hainan Affiliated Hospital of Hainan Medical University Institutional Review Board for Medical Ethics approved this retrospective analysis of anonymous data and waived the requirement for informed consent. Consecutive patients newly diagnosed with NPC in our hospital between April 2018 and December 2020 were included. The inclusion criteria were: (1) pathologically confirmed stage III–IV NPC; (2) application of pre-treatment DCE-MRI; (3) Karnofsky score ≥70; and (4) complete clinical and MR image data. The exclusion criteria were: (1) contraindications for MR examination; (2) poor imaging quality; (3) history of head or neck radiotherapy or chemotherapy; (4) other primary tumors; and (5) lost to follow-up.

All patients underwent MRI of the nasopharynx and neck, computed tomography (CT) scans of the chest, abdominal ultrasound or CT with enhancement, and bone scan. Patients at risk of distant metastases also underwent whole-body ^18^F-fluorodeoxyglucose positron emission tomography (PET)/CT. TNM status and clinical stage were determined according to the eighth edition of the International Union Against Cancer/American Joint Committee on Cancer (UICC/AJCC) staging system (24).

Patient baseline clinical characteristics and pathologic data were obtained from their medical records and included age, sex, Epstein–Barr virus (EBV), clinical stage, TNM stage, pathological type, HIF-1α, EGFR, Ki-67, tens of homolog deleted on chromosome ten (PTEN), and vascular endothelial growth factor (VEGF) protein expression.

### Treatment protocols

The treatment regimens were selected according to the NCCN guidelines (3). All enrolled patients underwent radical three-dimensional conformal and intensity-modulated radiation therapy (IMRT) with the following dose distribution: nasopharyngeal tumor volume (GTVnx) and cervical lymph node volume (GTVnd), 66–73 Gy and 63–70 Gy, respectively; high-risk area of primary focus (CTV1), 62–64 Gy; low-risk area of primary focus and cervical lymph node drainage area (CTV2), 54–58 Gy; and planning target volume (PTV1) and PTV2, 54 Gy and 50 Gy, respectively. All patients received one fraction per day for 5 days a week, with 31–35 segmentations. IC consisted of cisplatin and docetaxel and was administered at 75 mg/m^2^ by intravenous drip on day 1, or 25 mg/m^2^ by intravenous drip on days 1–3, every 3 weeks for 2–4 cycles. CCRT consisted of cisplatin 75 mg/m^2^ on day 1 (or 25 mg/m^2^ days 1-3) once every 3 weeks. NTZ treatment (200 mg weekly throughout radiotherapy) were performed when satisfy: (1) EGFR expression levels higher than 2+ staining patterns; (2) the patient’s preference; (3) the patient’s affordability.

### Follow-up and clinical endpoint

Patients were assessed every 3 months in the first 2 years, every 6 months in years 3–5, and then annually. The follow-up period was defined as the period from therapy initiation to the last clinical visit or death. The study endpoint was PFS, which was defined as the time from the date of treatment initiation to the date of the first disease recurrence, metastasis, death from any cause, or last follow-up, whichever came first. Disease progression was confirmed by biopsy pathology and/or imaging methods such as CT, MRI, or FDG-PET/CT.

### MRI protocols and analysis

For all patients, MRI was performed within 1 week before treatment. MRI was performed on a 3.0T MR scanner (Skyra, Siemens Medical Solutions, Erlangen, Germany) utilizing a 20-channel combined head and neck coil. The imaging protocol was as follows: (1) spin-echo (SE) sequence to obtain axial T1-weighted (T1W) images (repetition time [TR]=625 ms, echo time [TE]=9.0 ms, field of view [FOV]=180×180 mm^2^, matrix=256×256, and 4.0 mm slice thickness); (2) turbo spin-echo (TSE) sequence for the acquisition of axial proton density-weighted (PdW) images (TR=4070 ms, TE=30 ms, FOV=180×180 mm^2^, matrix=384×384, and 4.0 mm slice thickness); (3) fast low-angle shot (FLASH)/vibe sequence to obtain DCE including 50 dynamic acquisitions, 4.9 s per dynamic acquisition, with the following parameters: TR=4.09 ms, TE=1.47 ms, flip angle=9°, phase=75%, bandwidth=400 Hz, thickness=4 mm, slice gap=0 mm, FOV=180×180 mm^2^, matrix=192×144, TA=245 s; (4) FLASH/vibe sequence for T1-mapping before DCE-MRI with five different flip angles (3°, 6°, 9°, 12°, and 15°). Gadodiamide (Omniscan, GE Medical Systems, Amersham, Ireland) was injected intravenously (dosage, 0.1 mmol/kg; rate, 2 mL/sec followed by a 25-mL saline flush at 3.5 mL per second) *via* a power injector at the third dynamic acquisition.

DCE-MR images were analyzed using Omni-Kinetics software (GE Healthcare, China). The extended toft linear model was selected, using the intracranial internal carotid artery as the standard arterial input function (AIF) curve. The *K^trans^* (efflux rate constant), *κ_ep_* (reflux rate constant), *V_e_* (the extracellular extravascular volume), and *V_p_* (the intravascular plasma volume fraction) maps were automatically calculated.

### Tumor segmentation and radiomics feature extraction

A three-dimensional (3D) region of interest (ROI) covering the whole tumor was manually drawn on the DCE-MR images using ITK-SNAP (version 3.8.0, USA, http://www.itksnap.org) by a radiologist with 5 years of head and neck radiological diagnosis experience (Wenzhu Li) and was validated by a senior radiologist with 12 years of experience (Weiyuan Huang) who was blinded to the clinical and pathological information of the patients. The ROIs were then used to extract quantitative radiomics features for analysis. Intraclass correlation coefficient (ICC) values were applied to assess the stability and robustness of radiomics feature extraction. Fifty randomly selected patients were used to test the ICC. The radiological attending physician (Wenzhu Li) and the senior physician (Weiyuan Huang) independently delineated the ROI who were blinded to the clinical and pathological information of the patients. ICC values under 0.4, between 0.4 to 0.75, and above 0.75 indicated weak, moderate, and strong agreement respectively.

**Figure 1:**
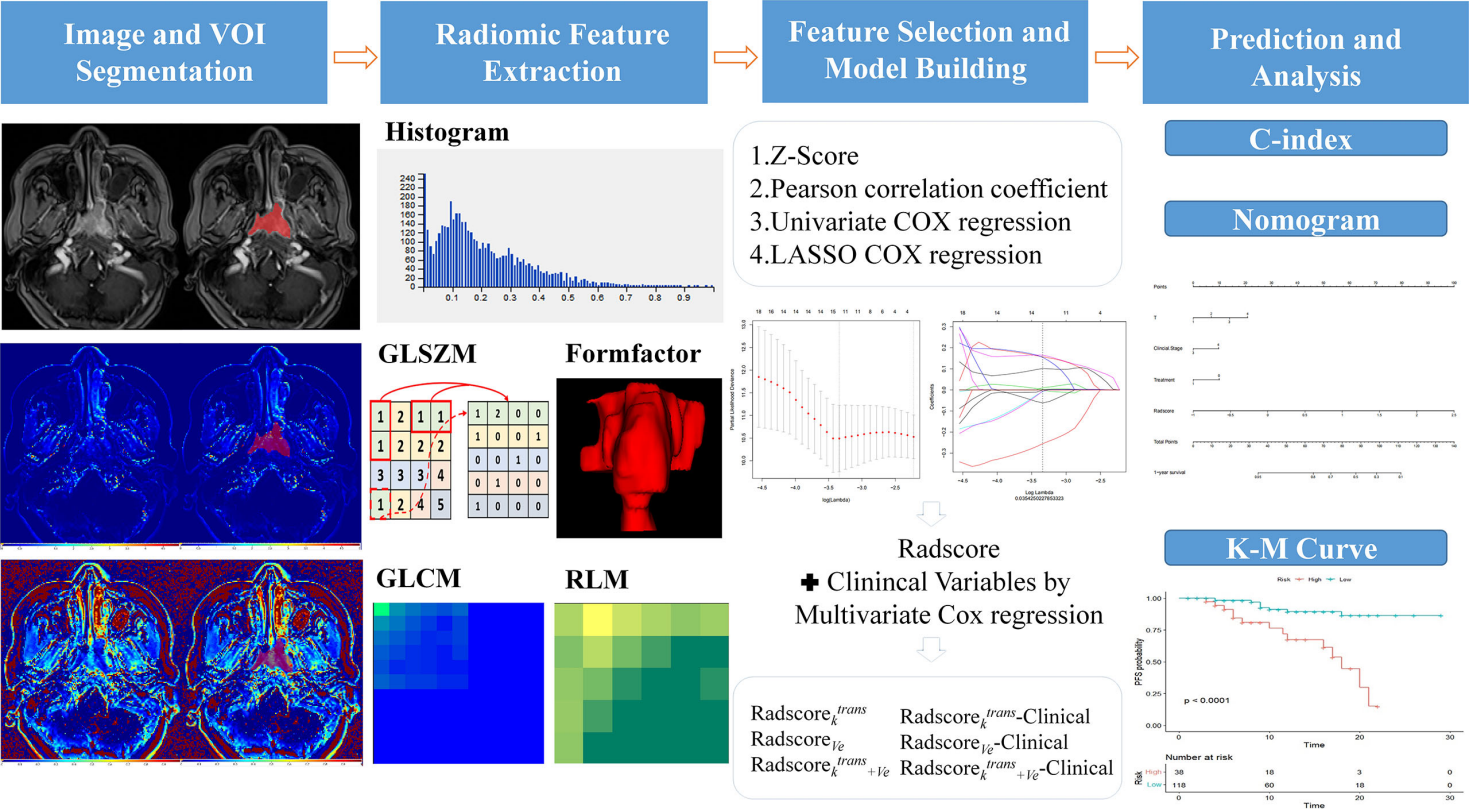
Workflow of radiomics analysis for predicting the prognosis of advanced nasopharyngeal carcinoma. The steps were: (1) Three-dimensional manual segmentation on *K^trans^* and *V_e_* images, (2) the calculation of six types of features for each patient from the defined segmentation, (3) LASSO Cox regression for feature selection and data dimension reduction, and (4) multivariate Cox regression to develop a radiomics nomogram model. Kaplan-Meier analyses were then performed to assess the prognostic value of the model. Abbreviations: VOI, volume of interest; GLSZM, gray-level size zone matrix; GLCM, gray-level co-occurrence matrix; RLM, run-length matrix; LASSO, least absolute shrinkage and selection operator; C-index, Harrell’s concordance index; K–M curve, Kaplan–Meier analysis curve.

The radiomic features of the lesions were extracted using AK software (Analysis Kit; GE Healthcare). A total of 360 features were extracted from *K^trans^* and *V_e _* mapping derived from DCE-MRI, including histogram features, gray-level size zone matrix (GLSZM) features, form factor features, gray-level co-occurrence matrix (GLCM) features, and run-length matrix (RLM) features. To eliminate the differences in the value scales of the extraction features, all features were normalized before selection. Each feature for all patients was normalized with Z-scores, subtracting the mean value and dividing by the standard deviation. Next, dimensionality reduction was performed on the data to eliminate those with similar characteristics after calculating the Pearson correlation coefficients. When the coefficient is larger than the threshold value (currently, the default is 0.9), one of them is removed randomly. This method ensures that the similarity of features after dimensionality reduction is not high. The radiomics workflow is shown in **Figure 1**.

### Model building and evaluation

To reduce the dimensionality and decrease redundant information, two steps were performed for feature selection. First, univariate Cox regression was performed for each feature. Features with *p*<0.05 were considered to be potentially associated with PFS and, thus, included in the following process. Least absolute shrinkage and selection operator (LASSO) Cox regression analysis was subsequently performed to identify the significant prognostic features, and 10-fold cross-validation was applied for parameters perfected and over-fitting reduction. LASSO achieves this by imposing a constraint on the model parameter (λ), which causes the regression coefficients of certain variables to shrink toward zero. All features with nonzero coefficients were selected in this step. A radiomics signature (Radscore) for each patient was built *via* a linear combination of selected features weighted by their corresponding non-zero coefficients. The Radscores of *K^trans^* images (Radscore*_k_^trans^*) *V_e_* images (Radscore*_Ve_*), and the combination of *K^trans^* and *V_e_* images (Radscore*_k_^trans^_+Ve_*) were then calculated for each patient. The clinicopathological characteristics of each patient were assessed using univariate Cox regression analysis.

To develop an optimal model, we developed a combination model (Radscore incorporating independent clinical predictors) using multivariable Cox regression analysis. Multicollinearity was assessed using variance inflation factor (VIF), and multicollinearity was considered when the VIF value exceeded 2. The VIF value was <2 indicating no multicollinearity among the predictors. Furthermore, a nomogram model was constructed based on Radscore and significant clinicopathological features to visualize the NPC prognosis. The optimal thresholds of the radiomics nomogram-defined score were identified using X-tile software (Yale University, New Haven, CT, USA). The patients were divided into high- or low-risk groups based on the score threshold. Kaplan–Meier analysis (log-rank tests) was used to analyze differences in PFS between the high- and low-risk groups. Harrell’s concordance index (C-index) was used to evaluate the discriminative ability of the prognostic models (Radscore, nomogram model) based on calculating the net benefit for patients at each threshold probability. Bootstrap analyses with 1,000 resamples were used to obtain C-index statistics that were corrected for potential overfitting. The value of the C-index ranges from 0.5 to 1.0, with 0.5 indicating random chance and 1.0 indicating perfectly accurate discrimination between the predicted probability and actual outcome.

### Statistical analysis

All statistical analyses were performed using *R* software (version 3.5.2, https://www.rproject.org). Univariate and multivariate Cox regression was performed using the “stats” package to identify independent prognostic factors. The “glmnet” package was used for LASSO Cox regression. The “survival” package was used for Kaplan–Meier analysis (log-rank tests) analyses. The “Rms” package was used to build the nomogram models. Harrell’s concordance index (C-index) was used to evaluate the nomogram models. X-tile (Yale University, New Haven, CT, USA) software was used to determine the optimal cut-off values of the radiomics nomogram-defined score. Statistical significance was set at *p*<0.05.

## Results

### Patient characteristics

A total of 217 consecutive patients with newly diagnosed stages III–IV NPC were identified in our hospital within the selected time frame. Sixty-one patients were excluded for images degraded by an artifact (n=14), incomplete immunohistochemical results (n=12), incomplete treatment (n=15), second primary tumor (n=6), and loss to follow-up (n=14). Thus, the analysis included 156 patients. Their detailed clinical information, histological characteristics, and treatment protocols are shown in **Table 1**. The median follow-up time was 12.4 months (range, 2–32 months) and the median PFS was 11.5 months (range, 2–29 months). During follow-up, 23 patients developed disease progression, including 6 local progression, 6 local recurrence, 3 distant metastases and 8 deaths.

**Table 1:** Characteristics of 156 patients with NPC.

	N (%)
Age(years)	49.99 (21-77)
Gender	
Male	118 (75.64%)
Female	38 (24.36%)
Pretreatment EB Virus	
Negative	48 (30.77%)
Positive	108 (69.23%)
Histology	
undifferentiated non-keratinized carcinoma	142 (91.03%)
differentiated non-keratinized carcinoma	13 (8.33%)
keratinized squamous cell carcinoma	1(0.06%)
Primary tumour staging	
T1	3 (1.92%)
T2	29 (18.59%)
T3	78 (50.00%)
T4	46 (29.49%)
Nodal staging	
N0	5 (3.21%)
N1	33 (21.15%)
N2	74 (47.44%)
N3	44 (28.21%)
Metastasis	
M0	138 (88.46%)
M1	4 (2.56%)
M_X_	14 (8.97%)
Clinial stage	
III	83 (53.21%)
IV	73 (46.79%)
Ki-67(%)	58% (10%-95%)
HIF-1α	
0	69 (44.23%)
1	51 (32.69%)
2	28 (17.95%)
3	8 (5.13%)
EGFR	
0	2 (1.28%)
1	21 (13.46%)
2	59 (37.82%)
3	74 (47.44%)
VEGF	
0	62 (39.74%)
1	73 (46.79%)
2	16 (10.26%)
3	5 (3.21%)
PTEN	
Negative	6 (3.85%)
Positive	150 (96.15%)
Nimotuzumab	
No	70 (44.87%)
Yes	86 (55.13%)

Ki-67 expression in tumor cells was semi-quantitatively scored based on the percentage of positively stained tumor cells.

HIF-1α: 0, no staining; 1, nuclear staining in 1–10% of cells; 2, nuclear staining in 11–50% of cells; and 3, nuclear staining in >50% of cells.

EGFR: 0,<10% of cancer cells with incomplete and weak cell membrane staining; 1, >10% of cancer cells showed incomplete and weak cell membrane staining; 2, >10% of cancer cells showed moderate cell membrane staining and<10% of cancer cells showed strong and intact cell membrane staining; and 3, >10% of infiltrating cancer cells showed strong, complete, and uniform cell membrane staining.

VEGF: 0, no staining; 1,<25% of the total number of positive cells; 2, 25–49% of the total number of positive cells; 3: >50% of the total number of positive cells.

NPC, nasopharyngeal carcinoma; HIF-1α, hypoxia-inducible factor-1α; EGFR, epidermal growth factor receptor.

### Feature selection and Radscore construction

**Figure 2:**
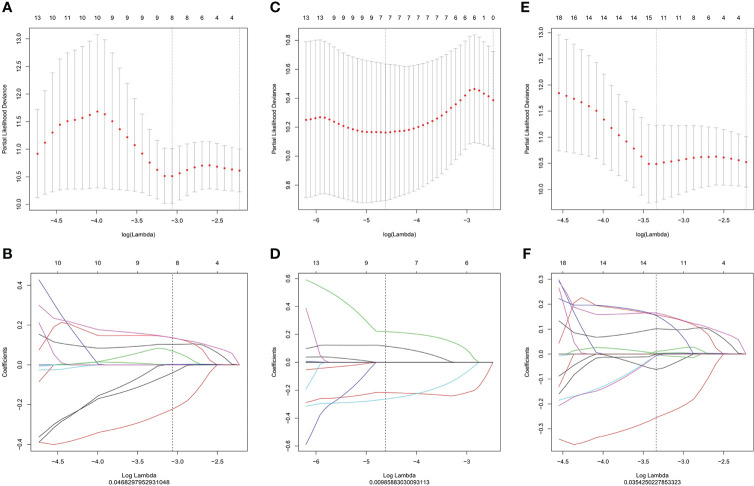
LASSO regression for radiomic feature selection in *K^trans^* **(A, B)**, *V_e_* images **(C, D)** and *K^trans^* +*V_e_* images **(E, F)**. **(A, C, E)** Selection of the optimal value of lambda (λ). Tuning log(λ) selection in the LASSO model used to perform 10-fold cross-validation *via* the minimum criteria. The y-axis indicates the partial likelihood deviance, while the lower x-axis indicates the log (λ) and the upper x-axis represents the average number of predictors. **(B)**, **(D)**, **(F)**: Each colored curve represents the trajectory of the change of an independent variable. Lambda values of 0.04682979 **(B)**, 0.00985883 **(D)**, and 0.03542502 **(F)** were selected as the optimal values, respectively. LASSO, Least absolute shrinkage and selection operator.

**Figure 3:**
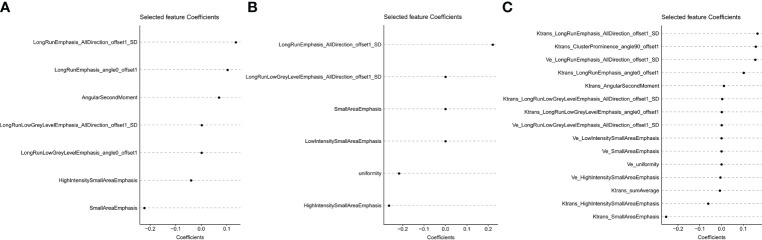
The x- and y-axes show the features and their coefficients, respectively. Finally, the 7, 6, and 15 features with non-zero coefficients were extracted from *K^trans^* **(A)**, *V_e _* **(B)**, and *K^trans^* +*V_e_* **(C)** images, respectively.

The ICC value of the *K^trans^* and *V_e_* maps was 0.990 (0.957, 0.994) and 0.993 (0.977, 0.996), respectively [median (lower quartile, upper quartile)]. Of the 360 radiomic features, 28 (7, 6, and 15 features extracted from *K^trans^*, *V_e_*, and *K^trans^* +*V_e_* images, respectively) were selected using the LASSO Cox regression model based on repeated 10-fold cross-validation (**Figure 2**). **Figure 3** shows the selected features. A radiomics signature was constructed using the selected features and their respective weights. The Radscore calculation formula consisting of these features is presented in the Supplementary Materials. The C-index was used to evaluate the predictive accuracy (discrimination) of the Radscore, the results of which are shown in **Table 2** Radscore*_k_^trans^_+Ve_* showed higher efficacy in predicting the PFS of patients with NPC compared to Radscore*_k_^trans^* and Radscore*_Ve_* Radscore*_k_^trans^* (hazard ratio [HR] 4.1263,95% confidence interval [CI] 2.2519–7.561, *p*<0.0001 Radscore*_Ve_* (HR 2.8549, 95%CI 1.7060–4.778, *p*<0.0001) and Radscore*_k_^trans^_+Ve_* (HR 3.5584, 95%CI 2.1341–5.933, *p*<0.0001) were independent prognostic factors associated with PFS in patients with NPC in multivariate Cox analyses, which indicated that patients with higher Radscores had a higher recurrence rate and poorer survival.

**Table 2:** C-indexes of the Radscore and Radscore-based models.

	C-index	95%CI
Radscore*_k_^trans^*	0.693	0.560~0.826
Radscore*_Ve_*	0.614	0.481~0.746
Radscore*_k_^trans^_+Ve_*	0.703	0.571~0.836
Radscore*_k_^trans^*-clinical	0.725	0.592~0.857
Radscore*_Ve_*−Clinical	0.689	0.548~0.813
Radscore*_k_^trans^_+Ve_*-clinical	0.732	0.599~0.864

C-index, index of probability of concordance; CI, confidence interval.

### Nomogram construction and evaluation

**Figure 4:**
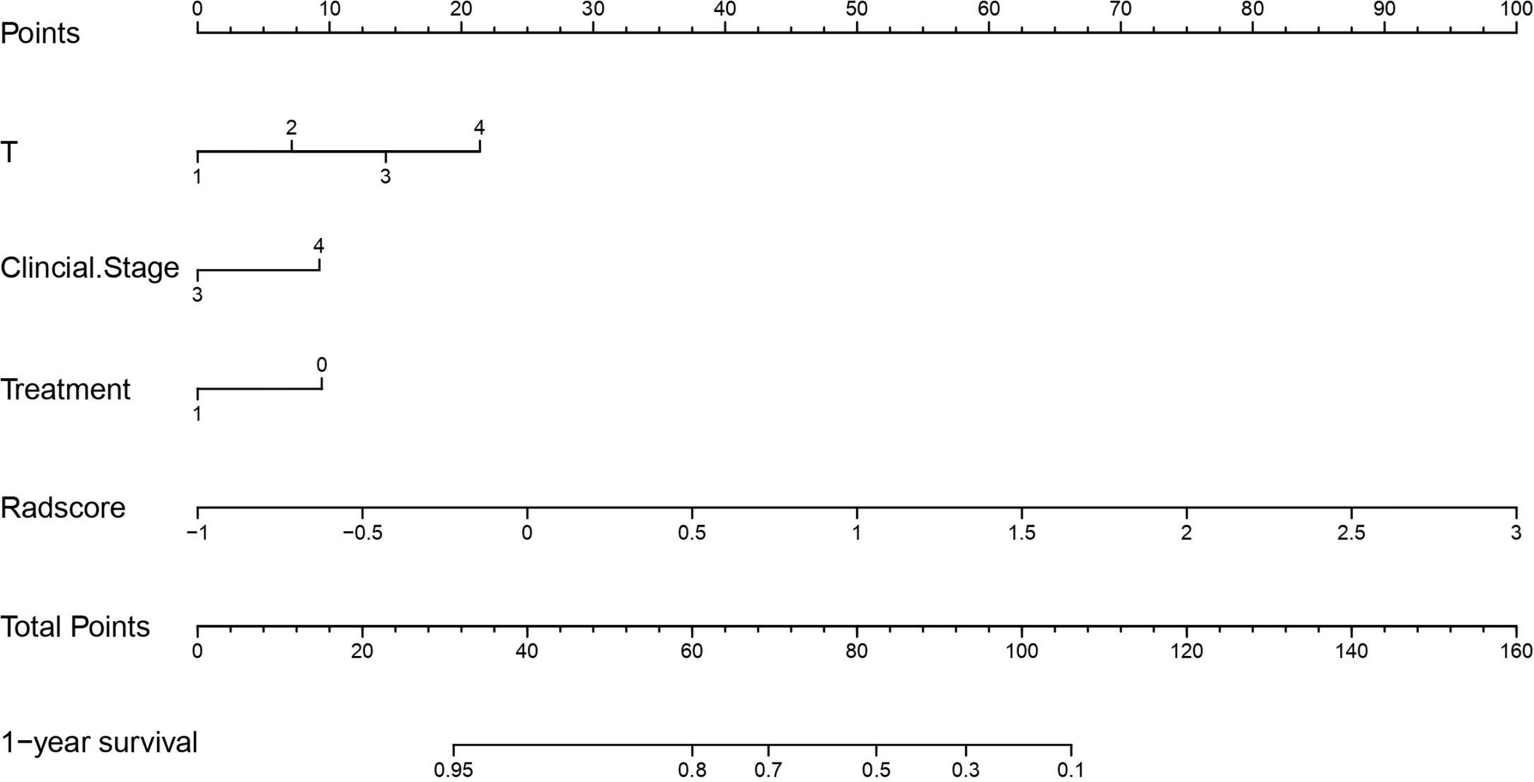
Radiomics nomogram for the prediction of progression-free survival in patients with advanced NPC. The radiomics nomogram was developed including the. Radscore*_k_^trans^_+Ve_* T stage, clinical stage, and treatment with NTZ incorporated. For each patient, the value of each variable is represented as points by projecting them onto the top line (point scale). Adding the points of all variables and projecting the total points value downward onto the bottom line can be used to calibrate the probability of 1-year progression-free survival. NPC, nasopharyngeal carcinoma.

**Figure 5:**
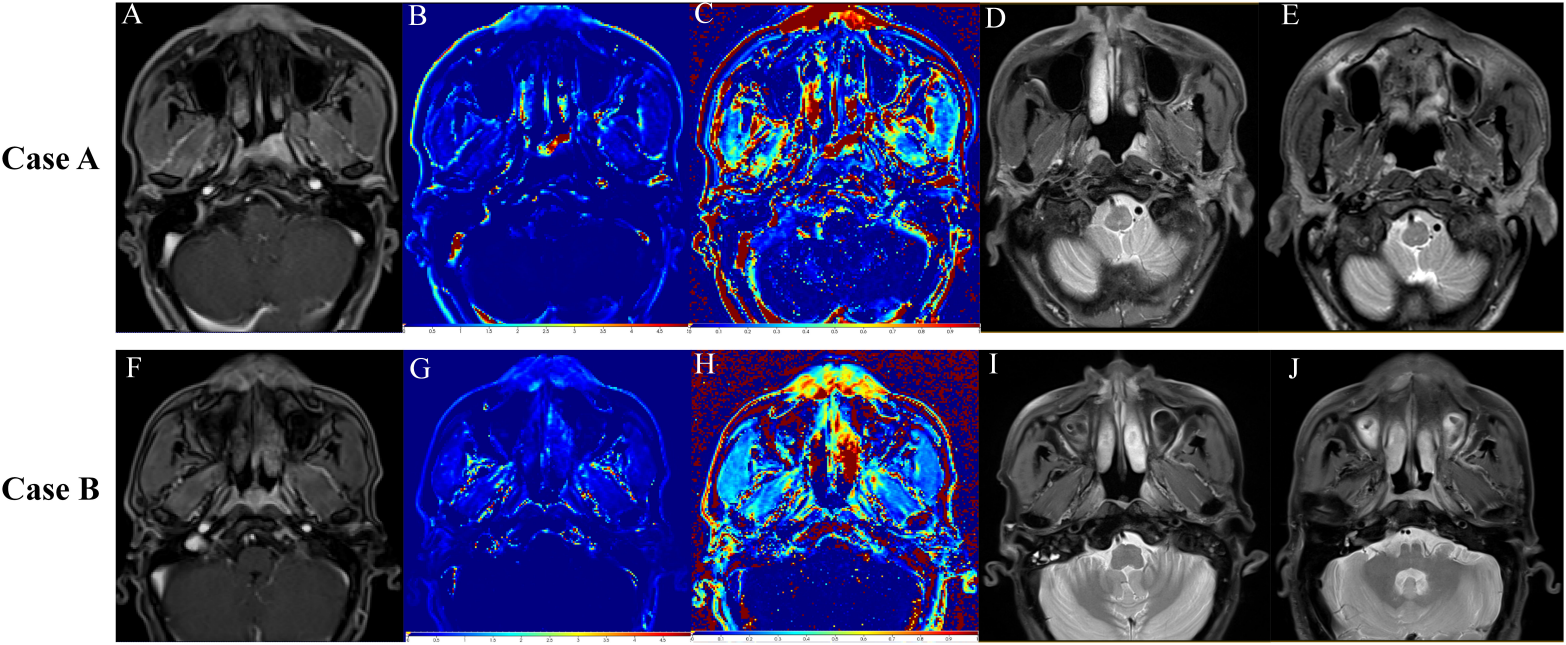
Representative images from three different time points during the follow-up period. The upper row shows images from Case A (T3N2M0) treated with nimotuzumab. **(A–C)** Baseline DCE-MR images. **(A)** Slice image. **(B)** *k^trans^* image. **(C)** *v_e_* image. **(D)** T2-weighted image at 3 months of follow-up showing shrinkage of the primary tumor lesion. **(E)** T2-weighted image at 23 months of follow-up showing complete regression of the primary tumor. The lower row shows images from Case B (T3N2M0) not treated with nimotuzumab. **(F–H)** Baseline DCE-MR images. **(F)** Slice image, **(G)** *k^trans^* image. **(H)** *v_e_* image. **(I)** T2-weighted image at 3 months of follow-up showing shrinkage of the primary tumor lesion. **(J)** T2-weighted image at 12 months of follow-up showing the first recurrence of the disease.

**Figure 6:**
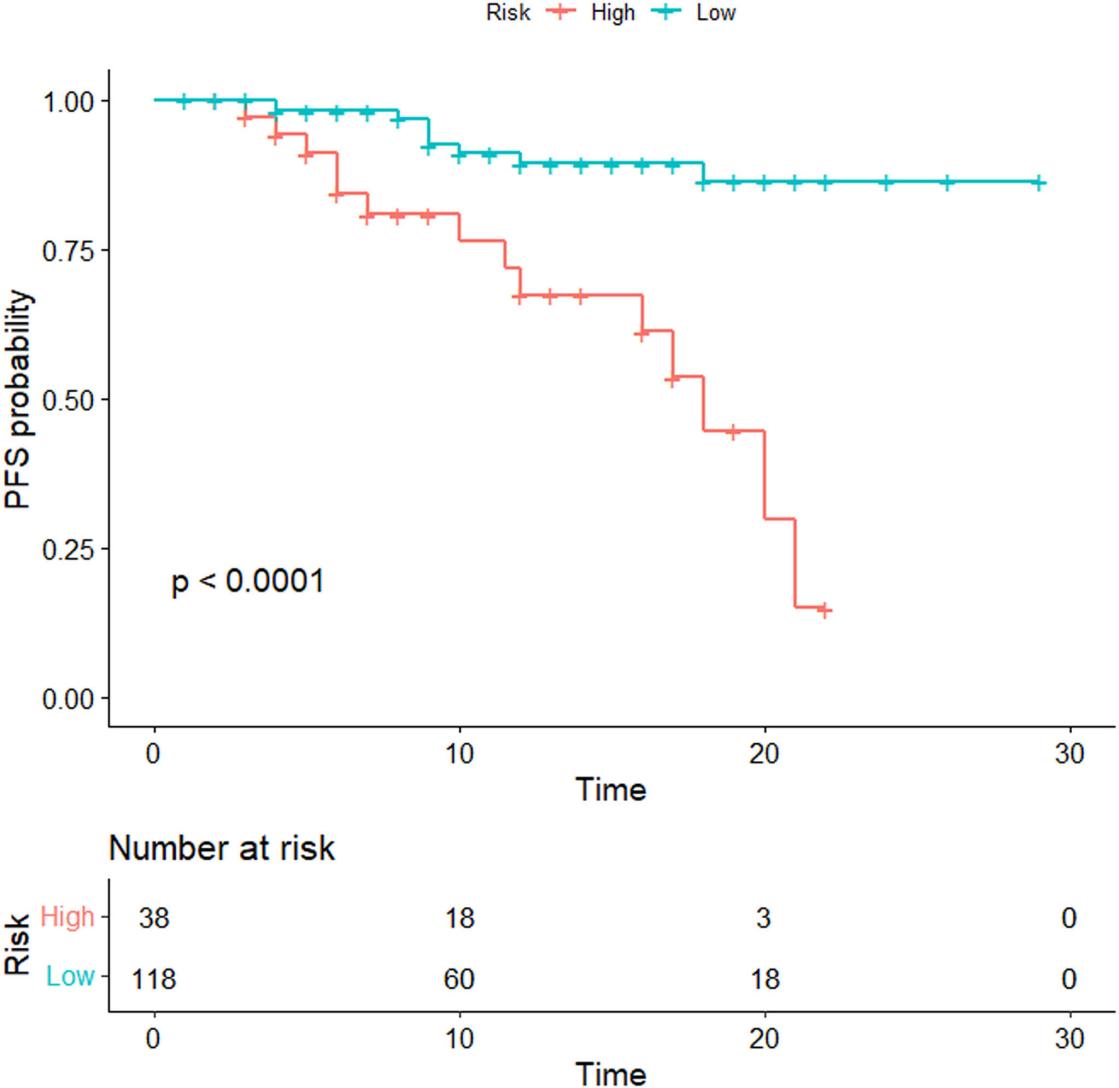
Kaplan–Meier analyses were performed to estimate progression-free survival in clinical subgroups. The patients were divided into high- and low-risk groups according to the threshold value of 3.1. Kaplan–Meier analysis showed a much lower PFS in the high-risk group than that in the low-risk group (*p* < 0.0001).

Univariate Cox regression analyses identified clinical stage, T-stage, and NTZ treatment as significant prognostic factors. The VIF values of clinical stage, T stage, NTZ treatment and Radscore*_k_^trans^_+Ve_* was 1.652, 1.674, 1.130 and 1.099 respectively, indicating no multicollinearity among the predictors. Therefore, three Radscores combined with T-stage, clinical stage, and NTZ treatment were utilized to develop the prediction models. The C-indices for the models are shown in **Table 2**. The Radscore*_k_^trans^_+Ve_*-clinical model yielded a maximum C-index of 0.732. Therefore, we developed the Radscore*_k_^trans^_+Ve_*-clinical nomogram (**Figure 4**). A lower Radscore*_k_^trans^_+Ve_* (HR 3.5584, 95%CI 2.1341–5.933), lower clinical stage (HR 1.5982, 95%CI 0.5262–4.854), lower T stage (HR 1.4365, 95%CI 0.6745–3.060), and receiving NTZ treatment (HR 0.7879, 95%CI 0.4899–1.267) were associated with longer PFS (**Figure 5**). Moreover, Radscore*_k_^trans^_+Ve_* had higher classification contributions compared to clinical variables in building the combined model. X-tile software identified an optimal Radscore*_k_^trans^_+Ve_*-clinical nomogram cutoff score of 3.1 for PFS prediction. Based on this threshold, the patients were assigned to high-risk (n=38, 24.36%) and low-risk (n=118, 75.64%) groups. Kaplan–Meier analysis showed a much lower PFS in the high-risk group than in the low-risk group (*p*<0.0001) (**Figure 6**).

## Discussion

This study developed a nomogram based on DCE-MRI-based radiomics, clinical stage, T-stage, and NTZ treatment to individually predict PFS in patients with advanced NPC. The radiomics features from the combination of *K^trans^* and *V_e_* images had better prognostic performance than those from either *K^trans^* or *V_e _* images alone. Integrating radiomics features with independent clinical factors adequately improved the predictive efficiency compared to the Radscore model. We then used the risk scores derived from the optimal model to classify patients into high- and low-risk groups, demonstrating its promise as a new biomarker for prognostic prediction in advanced NPC.

DCE-MRI allows the quantification of various vascular biomarkers that reflect the tumor microenvironment. It assesses the process of gadolinium leakage from the intravascular to extravascular compartments, which depends on multiple factors, including blood flow, vascular permeability, microvascular attenuation, and fractional volume of extracellular extravascular space (18). The *K^trans^* and *V_e_* of DCE-MRI quantitative parameters are well-established permeability parameters of tumors (25, 26). *K^trans^* stands for the amount of contrast agent transferred from blood to tissue and blood vessel permeability. *V_e_* refers to the amount of contrast agent every unit DCE-MRI allows. Previous studies reported that pre-treatment *K^trans^* and *V_e_* maps showed promise for predicting disease-specific survival and monitoring of treatment response in patients with NPC (19, 25, 26). In the present study, the multivariate Cox proportional hazards model suggested that the Radscore was the only independent prognostic biomarker. Radscores were calculated only using radiomic features derived from entire tumors on the DCE parameter maps. We confirmed that DCE is an effective approach for predicting the prognosis and treatment response of patients with NPC. In addition, several studies have demonstrated that tumor heterogeneity, which is associated with aggressiveness and treatment response, can be evaluated by texture analysis on the *K^trans^* and *V_e_* maps (27–29). Their results indicate that *K^trans^* and *V_e_* are more valuable in the evaluation of tumor heterogeneity than other DCE-MRI parameters. Similar to their findings, we also observed that *K^trans^* and *V_e_* showed good performance in predicting prognosis by non-invasively characterizing intra-tumor heterogeneity. Tumor heterogeneity may be associated with tumor angiogenesis, cell proliferation, necrosis, and even different tumor gene phenotypes (29). Higher tumor heterogeneity is highly associated with a poorer prognosis, which could be secondary to intrinsic aggressive biology or treatment resistance (30–32). Moreover, several studies have reported that radiomics features based on DCE-MRI may provide further insights into the heterogeneity of the tumor microvasculature and allow monitoring of pathophysiologic changes in various aspects of tumor vascular structure and functionality to help in evaluating tumor prognosis (23, 28, 33). Furthermore, our results also demonstrated the significant association of DCE-MRI-based radiomics with PFS in patients with NPC. Pak et al. (34) reported similar results for glioblastoma.

Our results showed that the radiomic combined model built on *K^trans^* and *V_e _* images demonstrated better prognostic performance than the models derived from *K^trans^* or *V_e_* images alone. Radiomic models based on multiple sequences showed greater efficiency than the model based on a single sequence (17, 35) as the combined model provides more morphological and functional information derived from multiple sequences rather than only a single sequence.

Subsequently, we integrated the Radscore*_k_^trans^_+Ve_* into a nomogram with clinical risk factors (clinical stage, T stage, and NTZ treatment) in patients with advanced NPC. Nomograms provide a scoring system and a visual prediction tool to help physicians rapidly evaluate individual survival post-treatment *via* a simple calculation in clinical practice. Yang et al. (4)established a nomogram that included radiomics, overall stage, and other clinical factors to predict PFS in locoregionally advanced NPC, with a C-index in the validation cohort of 0.811. Consistent with their findings, we also confirmed that the Radscore*_k_^trans^_+Ve_*-clinical nomogram was highly efficient in predicting PFS in advanced NPC. The C-index of the Radscore*_k_^trans^_+Ve_*-clinical nomogram in our study was 0.732 (95%CI: 0.599–0.864). This is the first attempt to include NTZ treatment as a risk factor in the nomogram to predict PFS in advanced NPC. In addition, our results demonstrated the association of advanced NPC treated with NTZ treatment and longer PFS.

Furthermore, we divided patients into high- and low-risk groups based on the optimal thresholds of the radiomics nomogram-defined score. The Kaplan–Meier survival curves showed a much lower PFS in the high-risk group than that in the low-risk group, which indicated that the radiomics nomogram may contribute to the precise stratification of patients for individual therapeutic strategies in clinical practice, thereby improving the clinical outcomes of patients with advanced NPC.

This study has some limitations. First, the sample size was not large enough for validation in an independent cohort. Nevertheless, we performed bootstrap validation, which provides a stringent assessment, and the model demonstrated good accuracy for predicting PFS. Second, the mean follow-up period was relatively short and a longer follow-up period was required to predict the 5-year PFS rates. Third, based on the NCCN guidelines, patients were treated with radiotherapy alone or CCRT and chemotherapy using varying doses of radiotherapy. This might be a confounding factor in the evaluation of PFS. Finally, this study did not further predict treatment response to NTZ in patients with advanced NPC due to the imbalance of sample size in the prognosis grouping. Future NPC studies will develop a new model based on pretreatment radiomics features to predict the survival benefit of NTZ for individual patients with NPC.

## Conclusion

The present study developed a nomogram to estimate individual PFS in patients with advanced NPC (stage III–IVb). The nomogram successfully stratified individual patients into high- and low-risk groups with distinguishable prognoses, which may provide a non-invasive and effective tool for prognostic prediction and risk stratification. Moreover, the nomogram also provided additional information on the association between longer PFS in patients with advanced NPC receiving NTZ treatment.

## Data availability statement

The original contributions presented in the study are included in the article/**Supplementary Material**. Further inquiries can be directed to the corresponding authors.

## Ethics statement

This study was reviewed and approved by The Hainan Affiliated Hospital of Hainan Medical University Institutional Review Board for Medical Ethics approved this retrospective analysis of anonymous data and waived the requirement for informed consent. Written informed consent for participation was not required for this study in accordance with the national legislation and the institutional requirements.

## Author contributions

WL, GW, FC and WH collected and analyzed the literature, drafted the figures and wrote the paper. TL, GD and QY performed the data collection. YL performed the statistical analysis. All authors contributed to the article and approved the submitted version.

## Funding

This work was supported by the Natural Science Foundation of Hainan Province [grant number 821MS125], the National Natural Science Foundation of China [grant number 81871346], National Natural Science Fund Cultivating 530 Project of Hainan General Hospital [grant number 2021QNXM18], and the Natural Science Research Project “open competition mechanism” of Hainan Medical College [grant number JBGS202113]. This project was supported by the Hainan Province Clinical Medical Center.

## Acknowledgments

We would like to thank all the patients and the personnel involved in this study.

## Conflict of interest

Author YL was employed by GE Healthcare.

The remaining authors declare that the research was conducted in the absence of any commercial or financial relationships that could be construed as a potential conflict of interest.

## Publisher’s note

All claims expressed in this article are solely those of the authors and do not necessarily represent those of their affiliated organizations, or those of the publisher, the editors and the reviewers. Any product that may be evaluated in this article, or claim that may be made by its manufacturer, is not guaranteed or endorsed by the publisher.
