# Supplementary material for: Dynamic contrast-enhanced magnetic resonance imaging-based radiomics for the prediction of progression-free survival in advanced nasopharyngeal carcinoma
Source: Front Oncol. 2022 Oct 13;12:955866. doi: 10.3389/fonc.2022.955866 (PMC9627984; doi:10.3389/fonc.2022.955866)
Supplement: Supplementary file 1 [file DataSheet_1.docx]

Supplementary Material

# Supplementary

Radscore*_K_^trans^*=0.07*AngularSecondMoment+0.135*LongRunEmphasis_AllDirection_offset1_SD+0.103*LongRunEmphasis_angle0_offset1+0.002*LongRunLowGreyLevelEmphasis_AllDirection_offset1_SD+0.001*LongRunLowGreyLevelEmphasis_angle0_offset1+-0.04*

HighIntensitySmallAreaEmphasis+-0.223*SmallAreaEmphasis+ 0.136

Radscore*_Ve_* =-0.225*uniformity+0.214*LongRunEmphasis_AllDirection_offset1

_SD+0*LongRunLowGreyLevelEmphasis_AllDirection_offset1_SD+-0.233*

HighIntensitySmallAreaEmphasis+0*LowIntensitySmallAreaEmphasis+0*SmallAreaEmphasis+ 0.09

Radscore*_K_^trans^ _+Ve_*=0.157**K^trans^*_ClusterProminence_angle90_offset1+0.011* *K^trans^* _

AngularSecondMoment+-0.008* *K^trans^* _sumAverage+0.165* *K^trans^* _

LongRunEmphasis_AllDirection_offset1_SD+0.102**K^trans^*_LongRunEmphasis_angle0_offset1+0.003**K^trans^*_LongRunLowGreyLevelEmphasis_AllDirection_offset1_SD+0.001* *K^trans^* _LongRunLowGreyLevelEmphasis_angle0_offset1+-0.061**K^trans^* _HighIntensitySmallAreaEmphasis+-0.255* *K^trans^* _SmallAreaEmphasis

+0**V_e_*_uniformity+0.155* *V_e_* _LongRunEmphasis_AllDirection_offset1_SD+0.001* *V_e_* _LongRunLowGreyLevelEmphasis_AllDirection_offset1_SD+-0.005* *V_e_* _

HighIntensitySmallAreaEmphasis+0* *V_e_* _LowIntensitySmallAreaEmphasis+0* *V_e_* _SmallAreaEmphasis + 0
